# Kalirin12 interacts with dynamin

**DOI:** 10.1186/1471-2202-10-61

**Published:** 2009-06-17

**Authors:** Xiaonan Xin, Chana A Rabiner, Richard E Mains, Betty A Eipper

**Affiliations:** 1 Neuroscience Department, University of Connecticut Health Center, Farmington, USA; 2 Department of Health and Human Services, Bethesda, MD, USA

## Abstract

**Background:**

Guanine nucleotide exchange factors (GEFs) and their target Rho GTPases regulate cytoskeletal changes and membrane trafficking. Dynamin, a large force-generating GTPase, plays an essential role in membrane tubulation and fission in cells. Kalirin12, a neuronal RhoGEF, is found in growth cones early in development and in dendritic spines later in development.

**Results:**

The IgFn domain of Kalirin12, not present in other Kalirin isoforms, binds dynamin1 and dynamin2. An inactivating mutation in the GTPase domain of dynamin diminishes this interaction and the isolated GTPase domain of dynamin retains the ability to bind Kalirin12. Co-immunoprecipitation demonstrates an interaction of Kalirin12 and dynamin2 in embryonic brain. Purified recombinant Kalirin-IgFn domain inhibits the ability of purified rat brain dynamin to oligomerize in response to the presence of liposomes containing phosphatidylinositol-4,5-bisphosphate. Consistent with this, expression of exogenous Kalirin12 or its IgFn domain in PC12 cells disrupts clathrin-mediated transferrin endocytosis. Similarly, expression of exogenous Kalirin12 disrupts transferrin endocytosis in cortical neurons. Expression of Kalirin7, a shorter isoform which lacks the IgFn domain, was previously shown to inhibit clathrin-mediated endocytosis; the GTPase domain of dynamin does not interact with Kalirin7.

**Conclusion:**

Kalirin12 may play a role in coordinating Rho GTPase-mediated changes in the actin cytoskeleton with dynamin-mediated changes in membrane trafficking.

## Background

**Figure 1:**
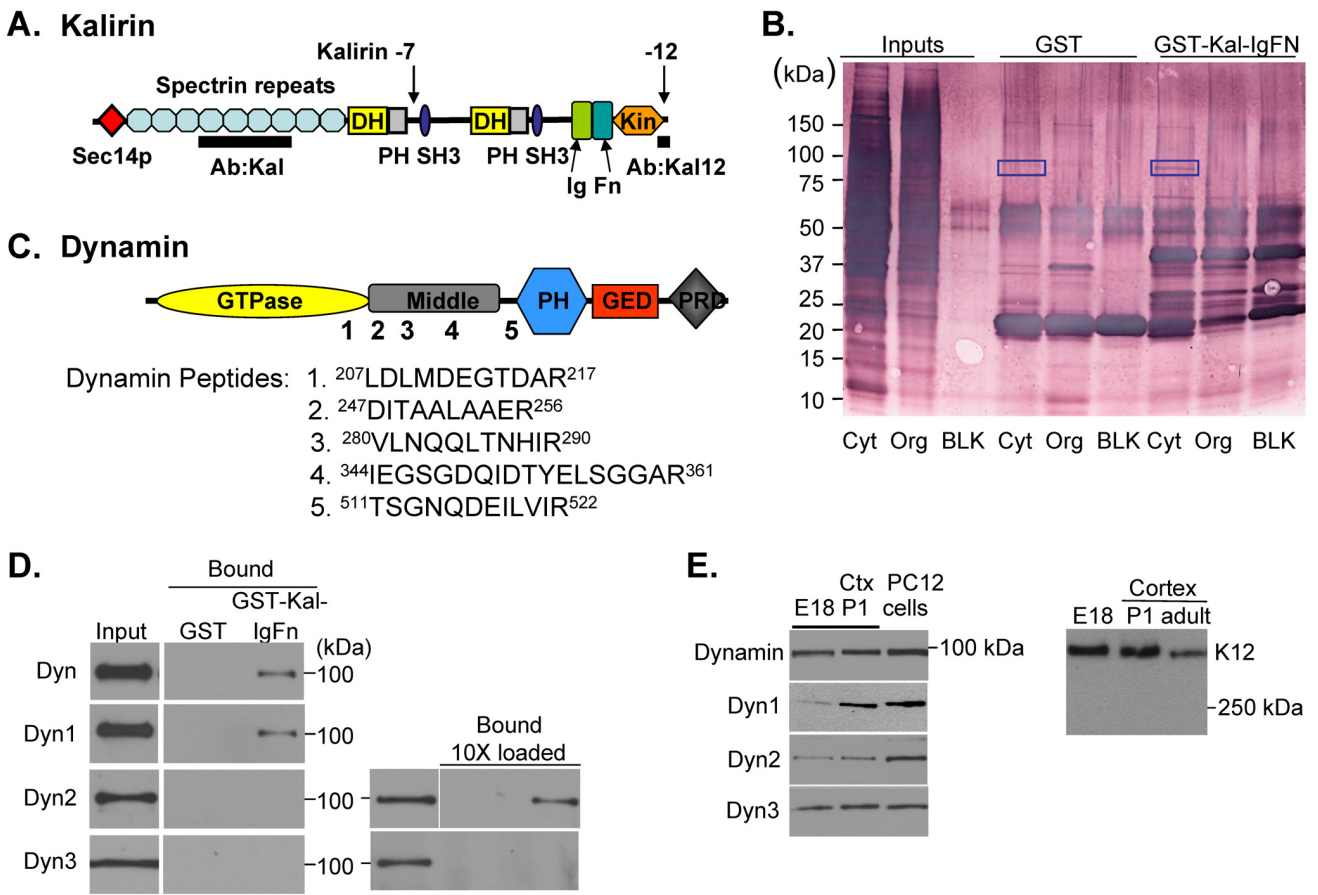
**The IgFn domain of Kalirin12 interacts with dynamin**. **A**. The domains of Kalirin12 are shown: Dbl homology (DH); pleckstrin homology (PH); Src homology 3 (SH3); immunoglobulin (Ig); fibronectin III (Fn); the alternate C-terminus of Kalirin7 is shown. The specificities of the spectrin-directed (Kal) and Kalirin12 antisera are indicated. **B**. Cytosolic (Cyt) and solubilized organellar (Org) fractions (5 mg protein) from adult rat cortex were incubated with GST-IgFn beads; homogenization buffer (BLK) was analyzed as a control. Proteins bound to the beads were fractionated by SDS-PAGE, transferred to PVDF membranes and visualized with Aurodye Forte. The 100 kDa band bound specifically to GST-IgFn was sequenced (boxed). **C**. The domain structure of dynamin is illustrated: pleckstrin homology (PH); GTPase effector domain (GED); proline-rich domain (PRD). The approximate locations of the five tryptic peptides identified by MS-analysis are indicated. **D**. Adult cortical cytosol (2.0 mg protein) was incubated with GST-IgFn beads. Beads were washed and bound proteins eluted. Eluate equivalent to 200 μg of lysate protein was fractionated; input (20 μg protein) was analyzed for comparison. In addition to the pan-dynamin (Dyn) antibody, dynamin1 (Dyn1), 2 (Dyn2) and 3 (Dyn3) antibodies were used. **E**. Total dynamin, dynamins1, 2 and 3 and Kalirin12 (C-terminal antibody; **Fig. 1A**) were visualized in SDS lysates (20 μg protein) prepared from the tissues and cells indicated: E18, embryonic day 18 rat brain; P1, postnatal day 1 rat cortex (Ctx); adult rat cortex; PC12 cells.

The human genome encodes sixty-nine GDP/GTP exchange factors (GEFs) for small GTPases of the Rho subfamily [1,2]. All share the ability to remove GDP from target Rho proteins, allowing GTP to bind so that downstream effectors can be activated. In addition to having two RhoGEF domains, the Kalirin/Trio subfamily is unique in its use of multiple protein/protein and protein/lipid interaction modules (Fig. 1A). Kalirin7, the most prevalent isoform in adult brain, begins with a Sec14p domain, includes multiple spectrin-like repeats and ends with a PDZ binding motif. Kalirin7 is concentrated at the post-synaptic density (PSD) and is necessary for spine maturation, maintenance and function [3-7]. Kalirin12, the largest isoform, is most prevalent during embryonic development, but is also present in adult neurons [8,9].

Features unique to Kalirin12 include tandem Ig and Fn domains as well as a putative kinase domain (Fig. 1A). While the *Drosophila TRIO *gene encodes neither an Ig nor a Fn domain, *C. elegans UNC73*, the paralog of Kalirin and Trio, encodes tandem IgFn domains and mammalian *TRIO *genes encode a single Ig domain [10]. Both extracellular and intracellular IgFn domains are involved in protein-protein interactions [11,12]. To gain insight into roles unique to Kalirin12, we searched for proteins that interacted with its IgFn domain, and identified the GTPase domain of dynamin.

Dynamin, a GTPase that causes membrane tubulation and fission, plays an essential role in both exocytosis and endocytosis [13,14]. Self-assembly increases the GTPase activity of dynamin [15-17], as does the binding of dynamin to PIP2-containing lipid tubules [18]. Dynamin interacts with several SH3 domain proteins through its C-terminal proline-rich domain (PRD) and with itself via the interaction of its GTPase domain with its assembly or GTPase effector (GED) domain [14] (Fig. 1C). In developing neurons, both exocytosis and endocytosis are critical players in the deployment and retraction of membrane, and must be coordinated with the assembly and disassembly of filamentous actin and microtubules in order to promote directed neuronal growth [19-21]. In mature neurons, dendritic spines must coordinate exocytosis and endocytosis to respond rapidly to incoming stimuli with changes in shape and altered deployment of receptors [22,23].

Rho GEFs of the Trio/Kalirin family, with their ability to activate Rac and RhoG, participate in the membrane remodeling associated with growth cone extension and active dendritic spines [6,24-29]. UNC-73 regulates the subcellular localization of UNC-40, a Deleted in Colorectal Cancer (DCC) receptor homolog, to direct growth cone migration [28]. Trio8, a splice variant which lacks the C-terminal GEF2, Ig and kinase domains [10], modulates endosome dynamics and neurite elongation in Purkinje neurons [30]. In AtT-20 cells, both Kalirin and Trio affect secretion via the regulated pathway [29,31]. Expression of exogenous Kalirin7 in non-neuronal cells inhibits the uptake of transferrin [32]. This response requires the presence of the Sec14p domain, which binds phosphatidylinositol-(3,5)-bisphosphate and phosphatidylinositol-(3)-phosphate, and the spectrin-like repeats, which allow Kalirin to oligomerize.

## Methods

### Expression vectors

Fragments of rat Kalirin were cloned into the pEAK10 vector (Edge Biosystems, Gaithersburg, MD) with a His_6_-myc epitope tag at the NH_2_-terminus [33]. Enhanced green fluorescent protein (from pEGFP-N2; Clontech, Mountain View, CA) replaced residues L^1127^STHTS^1132 ^of Kalirin12 in the pEAK GFP-Kalirin12 vector [6]. The IgFn region of Kalirin (L^2456^LG ... GIS^2625^) was inserted into the pEGFP-N2 vector to make a GFP-IgFn fusion protein, and into pGEX-6P (Amersham Biosciences) to generate GST-IgFn. pEGFP-N1 vectors encoding GFP fused to the C-terminus of human dynamin2, dynamin2/K^44^A, dynamin2/ΔPRD (lacking the Pro-rich domain, P^747 ^to D^870^), and dynamin2 GED/PRD (containing both the GED and Pro-rich domains, S^618 ^to D^870^) were the kind gifts of Dr. Pietro De Camilli (Yale University) [17,34,35]. A vector encoding GFP fused to the C-terminus of the GTPase domain of human dynamin2 (M^1 ^GN...RPD^320^) was generated by subcloning a PCR amplified fragment flanked by HindIII and EcoR 1 sites into pEGFP-N2. All constructs were verified by DNA sequencing.

### GST fusion protein expression and purification

Bacteria (*E. coli *BL21/λDE3) were transformed with vectors encoding pGEX.GST-IgFn or GST-Amphiphysin2 SH3 (a gift from Dr. Pietro De Camilli, Yale University), grown to stationary phase in LB medium containing ampicillin, diluted 100-fold and grown to OD_600 _= 1.3 at 37C, induced with 0.4 mM IPTG and grown at 16C overnight. Pellets of cells were resuspended in PBS (50 mM NaH_2_PO_4_, pH 7.4, 150 mM NaCl, 1 mM dithiothreitol, 5 mM EDTA, 1 mM PMSF) and passed 3 times through a French Press. Supernatants from a 500 ml culture were tumbled with 0.5 ml glutathione-Sepharose at 4C for 4 h. Beads were washed twice with 10 vol PBS, and once with 10 vol PBS containing 0.1% Triton X-100. Fusion proteins remained on the beads (stored at -80C). To elute the fusion proteins from the beads, beads were equilibrated with 10 vol of 50 mM Tris·HCl, pH 7.4, 150 mM NaCl, 1 mM DTT and eluted with 0.5 ml 50 mM Tris·HCl, pH 7.4, 150 mM NaCl, 1 mM DTT, 20 mM reduced glutathione. Fractions were dialyzed into 20 mM Hepes, pH 7.4, 100 mM NaCl, 1 mM DTT, 50% glycerol at 4C.

### GST-IgFn binding assay

Freshly dissected female adult rat cortex was minced and homogenized in 0.32 M sucrose, 10 mM Tris·HCl, pH 7.0 (10% w/v) using a Potter-Elvehjem homogenizer and nuclei were removed by centrifugation at 138 × g for 5 min. The supernatant was centrifuged at 435,000 × g, 15 min; the resulting supernatant constituted the cytosolic fraction. The high speed pellet was resuspended in 20 mM Na TES, 10 mM mannitol, 1% TX-100, pH 7.4 (TMT) with protease inhibitors for 30 min, then centrifuged at 435,000 × g, 15 min. The detergent supernatant is the solubilized organellar fraction; the insoluble pellet was discarded. Samples were pre-cleared by incubation with GST-glutathione-Sepharose 4B beads for 1 h, 4C and supernatants were incubated with GST-IgFn-glutathione-Sepharose 4B beads for 1 h, 4C and washed. Bound proteins were eluted by boiling in 1× SDS loading buffer; aliquots were fractionated on 4–20% polyacrylamide gels. Proteins transferred to PVDF membranes were visualized with Coomassie Brilliant Blue R250 or Aurodye Forte (Amersham Biosciences, Piscataway, NJ). For mass spectroscopy, bands were excised from Coomassie stained gels and prepared for trypsin digestion at the UCHC Proteomics & Biological Mass Spectrometry facility (Dr. David Han, Director) [36]. Five peptide sequences from rat dynamin1 were identified. Verification of mass spectroscopy results utilized antibodies that cross-react with all three dynamin gene products (pan-dynamin) (#610245, Pharmingen, San Diego, CA), or recognize individually dynamin1, 2, or 3 (#3456–3458, AbCam, Cambridge, UK).

pEAK Rapid cells were transiently transfected with vectors encoding EGFP or various fragments of dynamin. After 24 h, cells were extracted into 0.5 ml 20 mM NaTES, 10 mM mannitol, pH 7.4 (TM) containing protease inhibitor cocktail [29] and 0.2 mM Na_4_VO_3_. Following centrifugation (15,000 × g for 15 min), lysates were tumbled with GST or GST-IgFn beads, 4C, 2 h. After three rinses with TMT, proteins eluted by boiling in SDS sample buffer were separated by SDS-PAGE.

### Immunoprecipitation

Rat cortical cytosolic fractions (as described above) and extracts of transfected pEAK Rapid cells were used for immunoprecipitations. pEAK Rapid cells extracted in TM were centrifuged at 435,000 × g for 1 h to remove particulates. Samples (100 μg protein for tissue extracts, or 1/3 of total sample for pEAK lysates) were incubated with antibodies to Kalirin (polyclonal antibody Kal-spectrin, Fig. 1A) [33], GFP (polyclonal antibody, Abcam), or dynamin (monoclonal antibody, BD Biosciences) for 2 h at 4C; immune complexes were isolated with Protein G or A Sepharose (Pharmacia Biotech, Uppsala, Sweden). Samples were fractionated on 4–20% polyacrylamide SDS gels and Western blot analysis was carried out with antibodies to Kalirin12, dynamin or GFP (clone N86/8, NeuroMAb, UC Davis, Davis, CA).

### Primary cortical cultures and nucleofection

Cortices from E19.5 to P0 rats were diced and dissociated using a papain dissociation system (Worthington Biochemical Corporation, NJ). Plasmids (10 μg) were introduced into dissociated cells (10^7^) by nucleofection using the Rat Neuron Nucleofector Kit, program O-003 (AMAXA GmbH, Germany). Nucleofected and control neurons (0.3 × 10^6^/well) were plated onto poly-L-lysine coated glass coverslips and incubated in Neurobasal medium containing 10% fetal calf serum for 2 to 4 h; the medium was then changed to Neurobasal medium supplemented with 2% B27, 2% fetal calf serum, 0.5 mM L-glutamine, 25 μM L-glutamic acid, 100 U/ml penicillin and 0.1 mg/ml streptomycin (Invitrogen, Carlsbad, CA). After 4 days *in vitro*, 50% of the medium was replaced with fresh medium.

### Immunocytochemistry

After 3 h *in vitro*, cortical neuron cultures were fixed with 4% formaldehyde in PBS, permeabilized and blocked [29]. Primary antibodies were applied for 2 h and secondary antibodies for 1 h at room temperature; coverslips were mounted using Prolong Gold Antifade Reagent (Invitrogen). Images were captured using a Zeiss LSM510-Meta confocal microscope. Kalirin12 was detected using affinity-purified rabbit antibody JH3226 (1:100) [33]; Cy3-labeled donkey anti-rabbit IgG (1:1000, Jackson lab., PA) was used for co-staining with FITC-phalloidin (1:500, Sigma) while Alexa488-labeled donkey anti-rabbit IgG (1:500, Invitrogen) was used for co-staining with pan-dynamin monoclonal antibody (1:100, BD Biosciences) using Cy3-labeled donkey anti-mouse IgG (1:1000, Jackson Labs). Specificity of the Kalirin12 antibody was verified by pre-incubation (diluted 10-fold in blocking solution) with antigenic peptide (10 μg/ml) at 4C for 30 min; no signal was seen when the blocked antibody was applied to cortical neurons (6 DIV). βIII tubulin was detected with a chicken monoclonal antibody (1:2000; Aves Labs, Tigard, Oregon) followed by Alexa633-labeled goat anti-chicken IgG.

### Endocytosis of transferrin

After 6 days *in vitro*, cortical neurons nucleofected with vector encoding Kalirin12 or EGFP were rinsed with serum-free DMEM for 5 min and incubated with 50 μg/ml AlexaFluor546-transferrin (T23364, Invitrogen) in serum-free DMEM for 5 min at 37C. Cells were quickly rinsed with serum-free DMEM and fixed with 4% formaldehyde (J.T. Baker, Phillipsburg, NJ) in PBS. Uptake of transferrin by PC12 cells was assessed in a similar manner. Removal of surface bound transferrin with a low pH wash (50 mM glycine, 100 mM NaCl, pH 5.0 for 10 min at 37C) did not change the answer. Kalirin12 positive neurons were identified using myc monoclonal antibody and FITC-tagged donkey anti-mouse IgG; neurons were identified by simultaneously visualizing βIII tubulin using chicken antibody (1:2000, Aves Labs) and Alexa633-goat-anti-chicken IgG (Invitrogen, CA). To quantify AlexaFluor546- transferrin uptake, images from EGFP or Kalirin12 positive neurons were acquired under identical conditions using a Zeiss LSM510 Meta confocal microscope. Once background fluorescence was defined, the cell soma was identified automatically using the 488 nm signal (myc or EGFP) and SimplePCI software (Compix, Inc., Sewickley, PA); total and mean red within the cell soma were measured for each transfected neuron. Significance was calculated using a two-tailed Student's T-test.

### Dynamin self-assembly assay

Adult rat brains (1 g wet weight) were homogenized in 10 ml of buffer A (20 mM Hepes, pH 7.4, 150 mM NaCl, 1 mM MgCl_2_, 1 mM EGTA, 1 mM DTT, 0.3 mg/ml PMSF, protease inhibitor cocktail [29] with 10 mM NaF and 2 mM Na_3_VO_4_). Triton X-100 was added (1%) and the sample clarified by centrifugation at 100,000 × g for 10 min. Rat brain supernatant (10 ml; 8 mg protein/ml) was incubated with 0.5 ml GST-amphiphysin-2/SH3 domain [18] (8 mg) bound to glutathione Sepharose 4B equilibrated with buffer A. After tumbling for 4 h at 4C, beads were washed 3 times with 10 bed volumes buffer A. Proteins were eluted twice with 1 ml buffer B (20 mM Pipes, pH 6.5, 1.2 M NaCl, 1 mM DTT, protease/phosphatase inhibitors). Pooled eluates were dialyzed into buffer C (20 mM Hepes, pH 7.4, 100 mM NaCl, 1 mM DTT, 50% glycerol with protease/phosphatase inhibitors). Based on SDS-PAGE, the pooled eluate (2.0 ml) contained 0.1 mg/ml dynamin. Liposomes were prepared with 10% phosphatidylinositol-4,5-bisphosphate (#840046, Avanti, Alabaster, AL) and 90% brain polar extract (#141101, Avanti) [37,38]. Organic solvent was removed using dry argon and a vacuum pump. The lipid film was re-hydrated with buffer G (40 mM Hepes, pH 7.4, 1 mM MgCl_2_, 5 mM KCl, 135 mM NaCl, 1 mM DTT with protease/phosphatase inhibitors) by vortexing continuously for 15 min. After three freeze/thaw cycles and repeated extrusion (21 times) through a 400 μm filter (#800282, Whatman, Clifton, NJ), the liposome suspension was centrifuged at 15,000 × g for 5 min; the supernatant, which contains liposomes ≤ 400 μm in diameter was used. The dynamin assembly assay was performed using purified dynamin pre-incubated or not with GST or GST-IgFn on ice for 1 h and incubated at room temperature for 10 min with or without fresh liposomes. Oligomerized dynamin was pelleted by centrifugation at 60,000 × g for 15 min. Supernatant and pellet fractions were recovered and equal aliquots were subjected to SDS-PAGE; dynamin in each fraction was determined by staining proteins transferred to PVDF membranes with Coomassie Brilliant Blue.

## Results

### The IgFn domain of Kalirin12 interacts with Dynamin

Alternative splicing at the 3'-end of the Kalirin gene generates transcripts encoding Kalirin7 and 12 plus additional isoforms not diagramed (Fig. 1A) [33]. Each longer Kalirin isoform contains additional domains and a unique C-terminus. The domains unique to the largest characterized isoform, Kalirin12, presumably contribute to its functions in the cell. To explore this possibility, a GST-IgFn fusion protein was purified, bound to glutathione Sepharose 4B, and incubated with cytosolic and solubilized organellar fractions prepared from adult rat cortex. Proteins that bound to GST-IgFn, but not to GST, and were not in the control fraction were of interest (Fig. 1B). A 100 kDa protein was the most prevalent band meeting these criteria and was subjected to trypsin digestion and mass spectrometry. Five dynamin peptides were identified, suggesting that dynamin might interact with Kalirin12 (Fig. 1C). Dynamins1, 2 and 3 share the domain structure illustrated in Fig. 1C; three of the peptides identified were unique to dynamin1, one was also present in dynamins2 and 3, and the fifth was common to dynamins1 and 3, but not dynamin2.

To determine which dynamins could be identified amongst the proteins bound to the IgFn domain of Kalirin12, binding was evaluated utilizing antibodies directed towards dynamins1, 2 and 3, each of which is expressed in the adult cortex (Fig. 1D). Both dynamin1 and dynamin2 were detected, although detection of dynamin2 required loading more of the bound fraction onto the gel. An interaction between the IgFn domain of Kalirin12 and dynamin3 was not detectable. As in the adult brain, all three isoforms of dynamin are expressed in embryonic day 18 brain and in post-natal day 1 cortex (Fig. 1E); all three isoforms of dynamin are also expressed in rat PC12 pheochromocytoma cells, one of the cell lines utilized for these studies. Kalirin12 expression declined with age, but was readily detected in adult rat cortex; PC12 cells were previously shown to express Kalirin12 [39]. Although it was apparent that only a small percentage of the total dynamin interacted with the IgFn domain of Kalirin12 and these experiments do not establish a direct interaction, the interaction is specific.

### Kalirin12 interacts with dynamin in vivo

**Figure 2:**
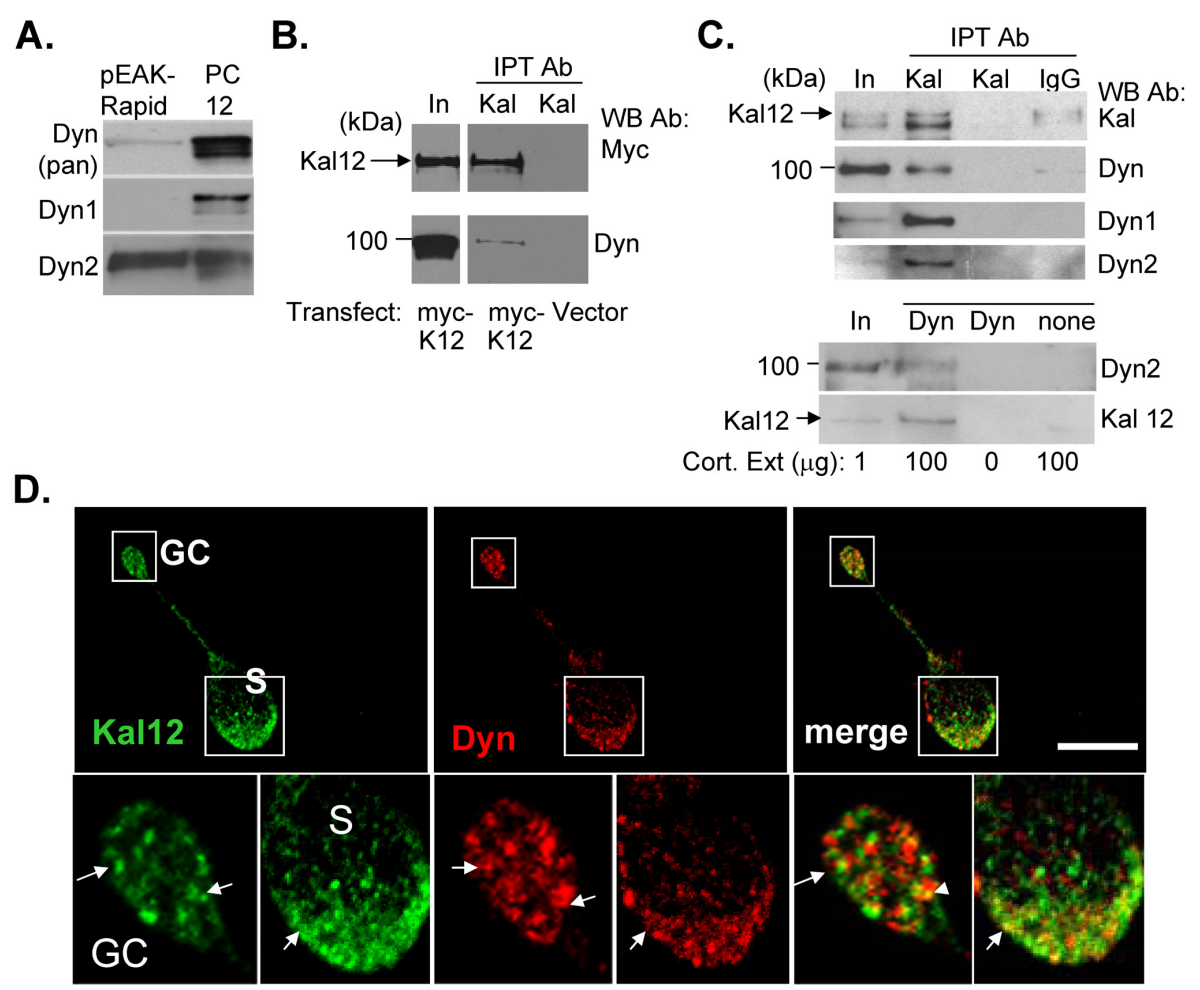
**Kalirin12 interacts with dynamin *in vitro *and *in vivo***. **A**. Lysates of pEAK Rapid and PC12 cells (20 μg protein) were blotted for total dynamin (pan), dynamin1 (Dyn1) or dynamin2 (Dyn2). **B**. pEAK Rapid cells were transiently transfected with pEAK vector encoding myc-Kalirin12 (myc-K12) or pEGFP-N2 (Vector). Extracts (100 μg protein) prepared 48 h later were immunoprecipitated with antibodies to Kalirin spectrin (Kal). Immunoprecipitation was verified using Myc antibody (upper) and co-immunoprecipitation was assessed with antibody specific for pan-dynamin (Dyn, lower); ratio of Input to Immunoprecipitated sample, 1:10. **C**. Cortical cytosol (Cort. Ext.; μg indicated below blots) was immunoprecipitated with Kalirin-spectrin antibody (Kal; upper) or dynamin antibody (Dyn; lower); controls included IgG or no antibody. Immunoprecipitated proteins were visualized with the same Kalirin antibody (upper) or dynamin2 antibody (lower). Co-immunoprecipitation was assessed using antisera to dynamin, dynamin1, dynamin2 or Kalirin12 as indicated; ratio of Input to Immunoprecipitated sample, 1:100. **D**. Primary cultures of cortical neurons were fixed 3 h after plating. Endogenous Kalirin12 was visualized using a rabbit polyclonal antibody for Kalirin12 and Alexa488-secondary (green). Endogenous dynamin was visualized using the pan-dynamin antibody (Dyn) and Cy3-secondary (red). S, soma; GC, growth cone; boxed regions are shown enlarged. White arrows, colocalized Kalirin12 and dynamin. Scale bar: 10 μm.

Since dynamin1 and 2 interacted with the isolated IgFn domain of Kalirin in a GST pull-down assay, we next evaluated their ability to interact with full-length Kalirin12 in intact cells. pEAK Rapid cells, a human embryonic kidney line, were transiently transfected with vectors encoding full-length myc-tagged Kalirin12. Based on Western blot analysis, these cells express dynamin2, but not dynamin1 (Fig. 2A), as expected for fibroblasts [40]. Cell extracts were immunoprecipitated with Kalirin antibody and analyzed with a pan-dynamin antibody (Fig. 2B). As observed in the GST pull-down assay, a small percentage of the endogenous dynamin was co-immunoprecipitated with Kalirin 12 (Fig. 2B).

We next wanted to determine whether endogenous Kalirin12 and dynamin interacted. Since Kalirin12 was enriched in embryonic brain, cytosolic fractions from rat embryonic cortex were analyzed. Immunoprecipitation with a Kalirin spectrin antibody and analysis with a pan-dynamin antibody demonstrated co-immunoprecipitation of dynamin (Fig. 2C, upper). Antisera specific for dynamin1 and dynamin2 were used to demonstrate co-immunoprecipitation of both dynamins with Kalirin (Fig. 2C, upper). The Kalirin spectrin antibody immunoprecipitates all of the major isoforms of Kalirin; to confirm the interaction of dynamin with Kalirin12, samples were immunoprecipitated with a pan-dynamin antibody and detected with a Kalirin12 specific antibody (Fig. 2C). Co-immunoprecipitation of dynamin and Kalirin12 was demonstrated.

We next compared the localization of Kalirin12 and dynamin in cortical neurons (Fig. 2D). When examined 3 h after plating, Kalirin12 positive puncta were prevalent in the soma (S) and growth cones (GC) of all cortical neurons. Pan-dynamin positive puncta were also apparent in the soma and in growth cones (Fig. 2D). Both proteins were present at multiple sites in these young neurons. Co-localization of a small fraction of the Kalirin12 and dynamin was observed in the cell soma and in growth cones (Fig. 2D, **enlarged box regions**). This result was consistent with the small fraction of the total dynamin or total Kalirin recovered by co-immunoprecipitation.

### The IgFn domain of Kalirin binds to the GTPase domain of dynamin in a GTP-dependent manner

**Figure 3:**
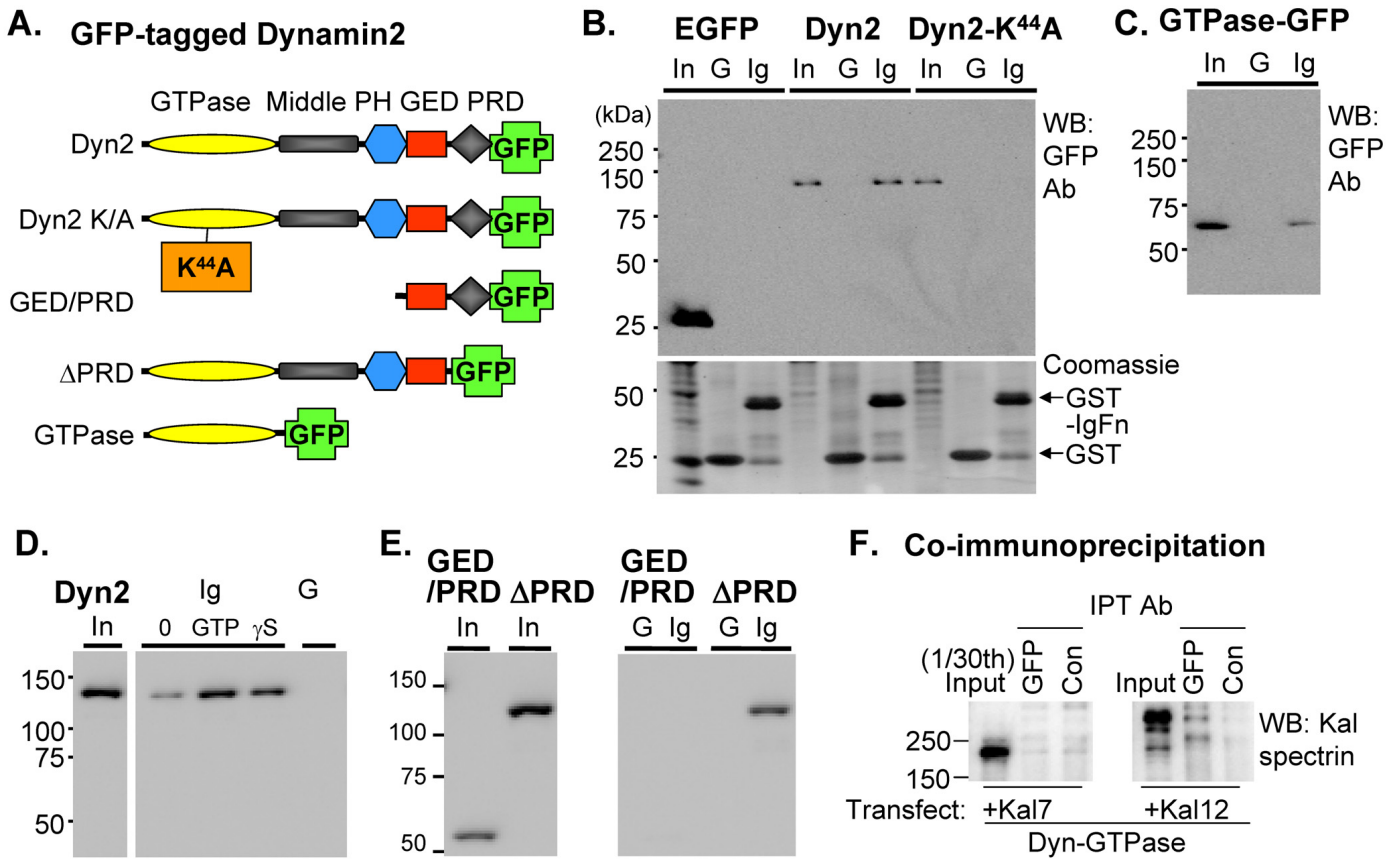
**The IgFn domain of Kalirin interacts with the GTPase domain of dynamin**. **A**. The GFP-tagged dynamin2 proteins expressed transiently in pEAK Rapid cells are shown diagrammatically; dynamin domains were defined in **Fig. 1C. B.–E**. Lysates were incubated with beads to which 2 μg GST (G) or 2 μg GST-IgFn (Ig) was bound. Bound proteins (50% of total) were eluted and fractionated on polyacrylamide gels; inputs (In; 1% of total) were analyzed simultaneously. EGFP and dynamin were visualized using antibody to GFP. **B**. Dynamin2 (Dyn2), but not dynamin2 K^44^A bound to GST-IgFn. The Coomassie Brilliant Blue image demonstrates similar amounts of GST and GST-IgFn. **C**. The GTPase domain of dynamin2 bound to Kalirin-IgFn. **D**. Binding of dynamin2 to GST-IgFn was enhanced by 1 mM GTP or 1 mM GTPγs. **E**. Dynamin2 lacking the PRD (ΔPRD) bound to Kalirin-IgFn while the GED/PRD domains of dynamin2 did not. **F**. pEAK Rapid cells expressing the dynamin2/GTPase-GFP fusion protein along with Kalirin7 or Kalirin12 were extracted and immunoprecipitated using a polyclonal GFP antibody or control immunoglobulin; the Kalirin-spectrin antibody detected an interaction of dynamin2 with Kalirin12, but not with Kalirin7.

The GTPase activity of dynamin is critical to its function [14,37]. Under physiological conditions, dynamins are loaded with GTP [14]. The ability of dynamin to bind GTP and function as a GTPase can be blocked by mutating the key Lys residue in the GTPase domain (Lys^44^) to Ala [41]. To determine whether the ability of Kalirin12 to bind to dynamin was sensitive to its GTP-binding state, lysates containing dynamin2-GFP or dynamin2/K^44^A-GFP (Fig. 3A) were incubated with beads containing GST or GST-IgFn (Fig. 3B). Dynamin2 was used for these studies because a collection of GFP-tagged variants were available [17,34,35]. While binding of dynamin2 was readily detected, dynamin2/K^44^A failed to bind to Kalirin IgFn (Fig. 3B). The presence of equivalent amounts of GST-IgFn and GST was verified by Coomassie staining (Fig. 3B, lower).

To determine whether the GTPase domain of dynamin bound to the IgFn domain of Kalirin, lysates containing dynamin2/GTPase domain were incubated with beads loaded with GST or GST-IgFn; binding of the isolated GTPase domain to the IgFn beads was readily detected (Fig. 3C). To explore further the effect of GTP on the interaction of dynamin with Kalirin-IgFn, GTP or GTPγS was added to lysates containing dynamin2/GFP before the GST-IgFn beads were added; both GTP and its non-hydrolyzable analog enhanced the interaction (Fig. 3D). In these crude lysates, addition of GTP could increase dynamin binding directly or indirectly, by affecting its oligomerization [42]. Consistent with the ability of dynamins1 and 2 to interact with Kalirin-IgFn, their GTPase domains are almost 90% identical (P21575; P39052).

The ability of other fragments of dynamin to bind to GST-IgFn was evaluated to determine whether additional interactions could be detected. The proline rich domain (PRD) at the C-terminus of dynamin (Fig. 3A) interacts with a number of proteins that contain Src-homology-3 (SH3) domains [14]. We expressed a fragment of dynamin2 containing only the GED and PRD and a truncated version of dynamin2 that lacked the PRD; both proteins were expressed well (Fig. 3A, E). While the GED/PRD fragment failed to bind to the IgFn domain of Kalirin, dynamin lacking its PRD (ΔPRD) continued to bind. Attempts to express the Middle/PH/GED or Middle domain fused to GFP led to insoluble aggregates.

We next wanted to determine whether isoforms of Kalirin that lacked the IgFn domain interacted with the GTPase domain of dynamin. The dynamin2/GTPase-GFP fusion protein and Kalirin7 or Kalirin12 were co-expressed in pEAK Rapid cells (Fig. 3F). The GTPase domain of dynamin2 interacted with Kalirin12, but not with Kalirin7. Taken together, our data indicate that the GTPase domain of dynamin binds to the IgFn domain of Kalirin in a GTP-dependent manner.

### The IgFn domain of Kalirin disrupts dynamin self-assembly

**Figure 4:**
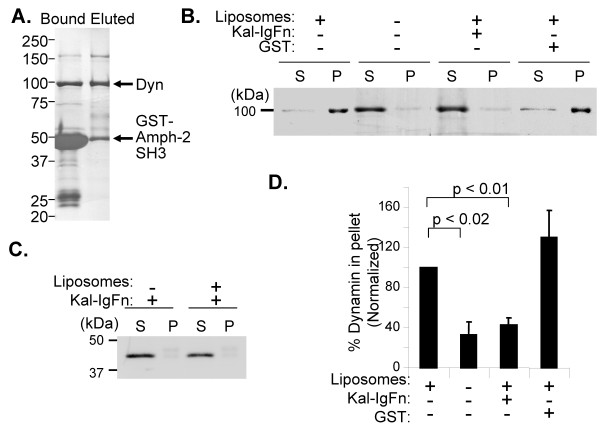
**Kalirin-IgFn domain disrupts dynamin self-assembly**. **A**. Dynamin purified from adult rat brain was fractionated by SDS-PAGE and visualized with Coomassie brilliant blue: Bound, aliquot of washed GST-amphiphysin2-SH3 beads incubated with rat brain cytosol; Eluted, aliquot of protein eluted by high salt/lower pH buffer. **B**. Purified dynamin (2.5 μg) was incubated on ice with or without purified GST (5 μg) or purified GST-IgFn (5 μg) for 1 h before exposure to liposomes, then incubated with freshly prepared liposomes for 10 min at room temperature; the control lacked liposomes. Liposomes were pelleted and supernatants and pellets were fractionated by SDS-PAGE; proteins were visualized by Coomassie Brilliant Blue staining the PVDF membrane. Supernatant lanes tend to be wider because of the glycerol-containing sample buffer used to prepare these more dilute samples. **C**. Purified GST-IgFn domain (5 μg) was incubated with or without liposomes (composed of 0.1 mM PIP2 and 0.9 mM brain polar lipids), without dynamin; Coomassie Brilliant Blue staining is shown. **D**. Coomassie stained dynamin in the supernatant and pellet was quantified using Gene Tools (Syngene, Frederick, MD). For each experiment (n = 4), dynamin in the "liposome only" pellet was set to 100%; the amount of dynamin in the other pellets was normalized to this value. Error bars are standard error of the mean; p values, two-tailed student T-test.

The GTPase activity of dynamin is increased dramatically following its oligomerization [14,16-18,37,42]. Phosphatidylinositol-4,5-bisphosphate-containing liposomes induce the oligomerization of purified rat brain dynamin, which can be assessed by quantifying the recovery of oligomerized dynamin from the liposome-containing particulate fraction [16,18]. The dynamin used for these studies was purified from adult rat brain using the SH3 domain of Amphiphysin2, which binds dynamin through its PRD (Fig. 4A); since adult rat brain was used, most of the dynamin in this preparation is dynamin1 [43]. In the presence of PI(4,5)P2-containing liposomes, Coomassie staining revealed the recovery of 97% of the dynamin from the particulate fraction; in the absence of liposomes, only 3% of the dynamin was recovered from the pellet (Fig. 4B). When purified, recombinant GST-IgFn domain was added to the mixture of PI(4,5)P2-containing liposomes and purified dynamin, most of the dynamin remained in the supernatant. The addition of GST did not have an inhibitory effect on dynamin oligomerization. To determine whether the IgFn domain of Kalirin bound to liposomes, thus indirectly blocking the binding of dynamin, IgFn domain was incubated with liposomes in the absence of dynamin. Coomassie blue staining revealed the presence of the IgFn domain in the supernatant fraction in the presence and absence of liposomes (Fig. 4C), indicating no direct interaction of the IgFn domain with liposomes.

Data from several experiments were quantified following normalization to the amount of dynamin pelleted in the presence of liposomes (Fig. 4D). The ability of liposomes to induce the oligomerization of purified dynamin was blocked by the addition of recombinant GST-IgFn domain (Fig. 4D). In these studies, the molar ratio of GST-IgFn:dynamin was approximately 5:1. Kalirin binds in a GTP-dependent manner to the GTPase domain of dynamin, blocking the ability of dynamin to oligomerize, and presumably to activate, upon interacting with phospholipids [18,42]. These data suggest an inhibitory role for Kalirin12 in regulating dynamin-mediated trafficking.

### Overexpression of Kalirin12 or IgFn disrupts endocytosis of transferrin in PC12 cells

**Figure 5:**
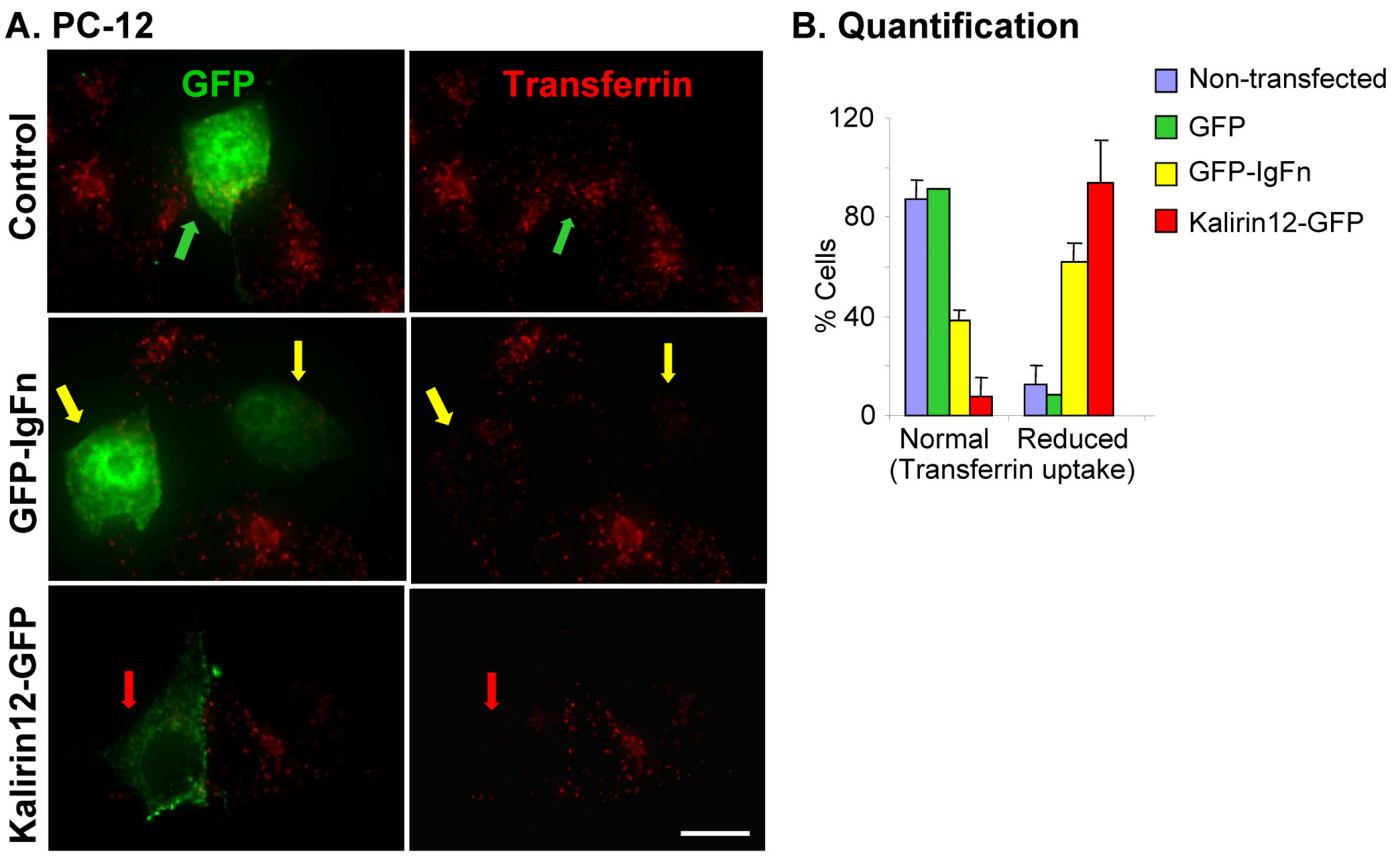
**Kalirin inhibits transferrin uptake by PC12 cells**. **A**. PC12 cells were transiently transfected with vectors encoding GFP (green arrows), GFP-IgFn (yellow arrows) or Kalirin12-GFP (red arrows). After 48 h, cells were fed with serum-free medium containing 50 μg/ml AlexaFluor546 transferrin; 10 min later, cells were rinsed in serum-free medium, fixed and analyzed. All images were taken using the same exposure time; scale bar, 10 μm. **B**. Endocytosis of transferrin was scored as normal if fluorescent puncta were readily detected in the interior of the cell and reduced if they were absent entirely or not centrally collected. Results are percentage of total number of transfected cells per group. Data are from three separate experiments. Total N for each group: non-transfected, 34; GFP, 24; GFP-IgFn, 25; Kalirin12-GFP, 20. Using chi square analysis, p < 0.001 for group distribution, GFP vs. GFP-IgFn, GFP vs Kalirin12-GFP and GFP-IgFn vs Kalirin12-GFP.

Dynamin oligomers constrict, severing the necks of nascent vesicles in a GTP-hydrolysis-dependent reaction [14,18,37]. This fission step is critical to the endocytic trafficking of many membrane proteins [13,44]. The uptake of fluorescently tagged transferrin provides an assessment of dynamin-mediated endocytosis [38]. To explore the possibility that Kalirin12 plays a role in controlling dynamin-mediated endocytosis, Kalirin12-GFP, its GFP-tagged IgFn domain or GFP were expressed in rat PC12 pheochromocytoma cells, which express both dynamin1 and dynamin2 (Fig. 1E). Following a 10 min incubation with fluorescently-tagged transferrin, cells were rinsed and fixed (Fig. 5A). Transferrin uptake by cells expressing GFP-IgFn (yellow arrows) was inhibited to varying extents compared to cells expressing GFP (green arrows); even when endocytosed by GFP-IgFn expressing cells, transferrin failed to accumulate in the perinuclear region, remaining distributed throughout the cell. PC12 cells expressing Kalirin12-GFP (red arrows) were unable to endocytose significant amounts of transferrin (Fig. 5A). Despite its lower levels of expression, Kalirin12-GFP had a more profound inhibitory effect on transferrin uptake than the isolated IgFn domain. Uptake of transferrin was categorized as Normal, with readily detected puncta of fluorescently-tagged transferrin accumulated in the interior of the cell, or Reduced (Fig. 5B). While 90% of non-transfected or GFP-transfected cells were categorized as Normal, endocytosis was categorized as Reduced in 60% of the cells expressing GFP-IgFn (Fig. 5B). Uptake was scored as Reduced in over 80% of the cells expressing Kalirin12-GFP.

### Overexpression of Kalirin12 disrupts endocytosis of transferrin in primary neurons

**Figure 6:**
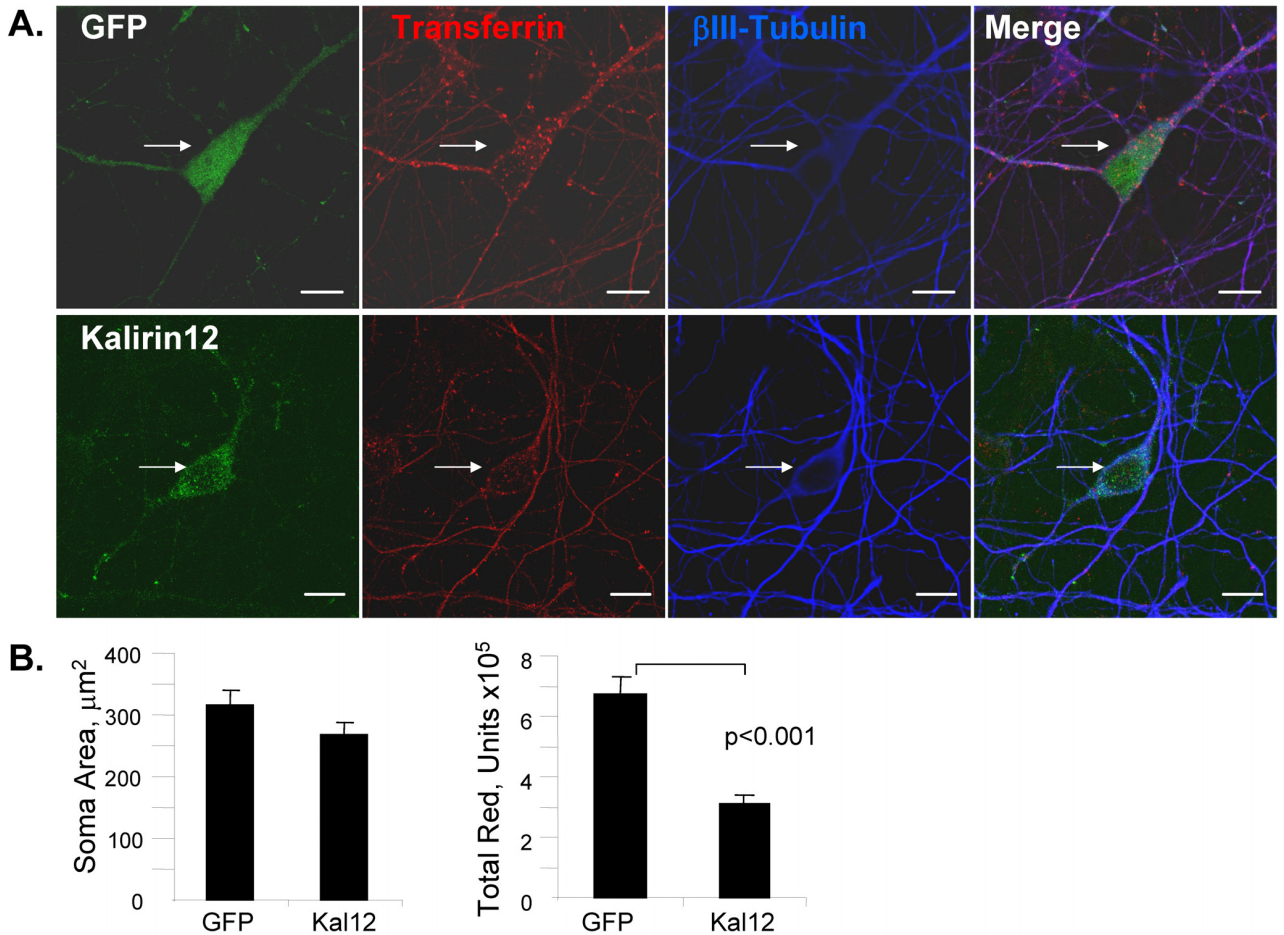
**Kalirin12 inhibits transferrin endocytosis in cultured cortical neurons**. **A**. Dissociated cortical neurons were nucleofected with vectors encoding GFP (upper) or myc-Kalirin12 (bottom) and examined after 6 days. AlexaFluor546-transferrin (red) was internalized for 5 min, cells were rinsed and fixed for visualization of Kalirin12 (myc; green) or GFP and βIII tubulin (blue). White arrows identify the cell soma. Scale bar, 10 μm. Including an acidic wash to remove surface transferrin did not alter the results. **B**. Transferrin signal in the cell soma was quantified in ≥ 20 images from GFP and Kalirin12 expressing neurons. Soma area and total red intensity were quantified. SEM is indicated; bracket, p < 0.001 using two-tailed student T-test.

We next explored the effects of exogenous Kalirin12 and IgFn on transferrin uptake by primary cultures of rat cortical neurons. After 6 days *in vitro*, cultures were incubated with AlexaFluor546-transferrin for 5 min, rinsed and fixed (Fig. 6A). Cells expressing EGFP or myc-Kalirin12 were located and identified as neurons based on staining for βIII tubulin. Internalized transferrin was quantified in at least 20 images by measuring total red intensity in the cell soma. Over-expression of Kalirin12 reduced total red intensity in the cell soma by approximately 50%; soma area was unaffected (Fig. 6B). In a separate series of experiments using postnatal day 1 striatal neurons, expression of GFP-IgFn or Kalirin12-GFP was found to inhibit transferrin uptake (data not shown). Although our co-immunoprecipitation data indicate that only a small percentage of the endogenous Kalirin12 and dynamin is stably interacting at any given time, our functional data demonstrate that over-expressed, exogenous Kalirin12 has a powerful inhibitory effect on dynamin-mediated endocytosis.

## Discussion

### IgFn domains distinguish isoforms of Kalirin/Trio

The IgFn domain of Kalirin binds to the GTPase domain of dynamin, inhibiting dynamin self-assembly. Although the *dTrio *gene does not encode IgFn domains, the *C. elegans unc-73 *gene generates isoforms that include tandem IgFn domains [10,45,46]. Mammalian Trio has a single Ig domain that binds RhoA, increasing its GEF activity [47]; RhoA does not bind to the IgFn domain of Kalirin (data not shown). The IgFn regions of mammalian Kalirin and Trio are more divergent than any other domain [9]. In addition to Kalirin12, only Duet, which lacks the spectrin-repeats and first GEF domain of Kalirin12, contains the tandem IgFn domains [10].

IgFn domains, with their 7 anti-parallel β-sheets, often serve as protein-protein interaction sites and are more common in extracellular than intracellular proteins [11,48,49]. Intracellular proteins with IgFn domains include titin, with 300 Ig domains, and short smooth muscle myosin light chain kinase, with three Ig domains and one Fn domain [11,49]. When mechanically stretched, the Ig and Fn domains of titin vary in the force required for unwinding, with Fn domains generally disrupted more easily than Ig domains. With Kalirin12 linked to plasma membrane phosphoinositides through its Sec14p domain [32] and to dynamin, it is conceivable that force-mediated unwinding of the IgFn domain could occur.

### Kalirin12 interacts with dynamin

Dynamin is crucial for membrane fusion and fission during exocytosis and endocytosis [14,35]. Our data indicate that the IgFn domain of Kalirin binds dynamin *in vitro *and *in vivo *(Figures 1 and 2), and in a GTP-dependent manner (Figure 3). Mutation of Lys^44^, a key residue in the GTP binding site of dynamin2 [41], reduced the binding of dynamin2 to Kalirin-IgFn. In the presence of GTP or GTPγS, the binding of dynamin2 to Kalirin-IgFn was increased. While Kalirin12 and Kalirin-IgFn bound to the 35 kDa GTPase domain of dynamin2, Kalirin7, which lacks the IgFn domain, did not (Figure 3).

Both dynamin and Kalirin are multiply phosphorylated and their interaction may be regulated by phosphorylation state [50-52]. Constitutive phosphorylation of dynamin is catalyzed by protein kinase C and Cdk5; along with synaptojanin, amphiphysin, and epsin, dynamin must be dephosphorylated before it can assemble and promote vesicle scission [52-54]. Kalirin is also phosphorylated by Cdk5 and rapidly dephosphorylated by protein phosphatase 1 under basal conditions [55]. Dynamin interacts with a variety of proteins through its C-terminal proline-rich domain, targeting it to specific sites of action [14]. Whether the two SH3 domains of Kalirin bind to the proline-rich domain of dynamin has not yet been investigated.

### The Kalirin-IgFn domain blocks dynamin self-assembly and inhibits dynamin-mediated endocytosis

Dynamin, through its GTPase activity, functions as a mechanoenzyme, facilitating membrane fission [14,34,37]. Self-assembly is an essential step in activating the GTPase activity of dynamin [15-17,42]. Using an *in vitro *assay [16] we demonstrated that the IgFn domain of Kalirin blocked liposome triggered dynamin self-assembly (Figure 4). Other proteins known to interact with the GTPase domain of dynamin in a GTP-dependent manner include the PHOX homology domain of phospholipase D, which acts as a GTPase activating protein (GAP) for dynamin [56]. The SH3 domain of phospholipase C-γ1 binds to the proline rich domain of dynamin1 and functions as a guanine nucleotide exchange factor (GEF) for dynamin1 [57]. The fact that exogenous Kalirin12 inhibited uptake of transferrin (Figures 5 and 6), a dynamin-mediated process, suggests that a similar interaction may be used normally in neurons. Tethered to the membrane via its Sec14p, PH or spectrin-like repeats and to dynamin via its IgFn domain, Kalirin12 could link activation of dynamin to membrane deformation and cytoskeletal reorganization.

### A role for Kalirin in endocytosis

Exocytosis and endocytosis, which are prevalent at growth cones [58], must occur in a carefully balanced and spatially controlled manner in response to multiple attractive and repulsive signals. Similarly, the insertion and retrieval of the receptors prevalent in dendritic spines requires carefully controlled, signal mediated exocytosis and endocytosis [22,59]. Kalirin12 is well equipped to play a key role in both processes. Kalirin12, like its *Drosophila *paralog, dTrio, is localized to vesicular structures and membranes in neuronal processes and growth cones [8,27]. In addition to a general role in endocytosis, the *C. elegans *paralog, UNC-73, may affect growth cone guidance by regulating the localization of UNC-40, a growth cone guidance cue receptor [28,45,60,61]. In the adult brain, expression of Kalirin7, which does not interact with the GTPase domain of dynamin, greatly exceeds expression of Kalirin12, but both proteins can be identified at the PSD [7]. The N-terminal region of Kalirin7, which is shared by Kalirin12, localizes to the sub-plasma membrane region of non-neuronal cells, where it inhibits the uptake of transferrin [7]. Both the phosphoinositide-binding Sec14p-domain of Kalirins7 and 12 and their spectrin-like repeat regions, which have been shown to interact with Arf6-GDP [62,63], could contribute to additional interactions affecting endocytic trafficking in dendritic spines and axonal terminals.

## Conclusion

The IgFn domain unique to Kalirin12 interacts with the GTPase domain of dynamin in a GTP-dependent manner and inhibits dynamin oligomerization. Transient interactions between dynamin and Kalirin12, with its N-terminal phosphatidylinositide binding Sec14p domain, multiple spectrin-like repeats and RhoGEF domains, may facilitate the coordination of endocytic trafficking and changes in the actin cytoskeleton.

## Authors' contributions

CAR identified dynamin as an interactor with the IgFn domain of Kalirin and carried out co-immunoprecipitation experiments confirming this interaction. XX demonstrated the GTPase domain of dynamin as the interacting region and performed studies with purified dynamin. Both CAR and XX used cultured neurons to examine the role of Kalirin in endocytosis. REM and BAE conceived of the study and assisted with performing experiments, data analysis and interpretation. All authors participated in writing the manuscript and approved its final form.
